# Use of Epidemiology Surge Support to Enhance Robustness and Expand Capacity of SARS-CoV-2 Pandemic Response, South Africa

**DOI:** 10.3201/eid2813.212522

**Published:** 2022-12

**Authors:** Rayna Taback-Esra, Diane Morof, Melissa Briggs-Hagen, Helen Savva, Simangele Mthethwa, Daniel Williams, Jennifer Drummond, Nancy Rothgerber, Michelle Smith, Meredith McMorrow, Mduduzi Ndlovu, Adeboye Adelekan, Gurpreet Kindra, Jacobus Olivier, Nkosi Mpofu, Katlego Motlhaoleng, Landiwe Khuzwayo, David Makapela, Patience Manjengwa, Anne Ochieng, Sarah Porter, Jonathan Grund, Karidia Diallo, Romel Lacson

**Affiliations:** US Centers for Disease Control and Prevention, Pretoria, South Africa (R. Taback-Esra, D. Morof, M. Briggs-Hagen, H. Savva, S. Mthethwa, D. Williams, J. Drummond, N. Rothgerber, M. Smith, M. McMorrow, M. Ndlovu, A. Adelekan, G. Kindra, J. Olivier, N. Mpofu, K. Motlhaoleng, L. Khuzwayo, D. Makapela, P. Manjengwa, A. Ochieng, S. Porter, J. Grund, K. Diallo, R. Lacson);; US Public Health Service Commissioned Corps, Rockville, Maryland, USA (D. Morof, M. Briggs-Hagen, M. McMorrow)

**Keywords:** COVID-19, coronavirus disease, SARS-CoV-2, severe acute respiratory syndrome coronavirus 2, viruses, respiratory infections, zoonoses, vaccine-preventable diseases, epidemiologic capacity, South Africa

## Abstract

As COVID-19 cases increased during the first weeks of the pandemic in South Africa, the National Institute of Communicable Diseases requested assistance with epidemiologic and surveillance expertise from the US Centers for Disease Control and Prevention South Africa. By leveraging its existing relationship with the National Institute of Communicable Diseases for >2 months, the US Centers for Disease Control and Prevention South Africa supported data capture and file organization, data quality reviews, data analytics, laboratory strengthening, and the development and review of COVID-19 guidance This case study provides an account of the resources and the technical, logistical, and organizational capacity leveraged to support a rapid response to the COVID-19 pandemic in South Africa.

SARS-CoV-2 was identified in late December 2019, the first cases occurring in Wuhan, China (*1*). A rapid spread of the virus in China, followed by an exponential increase of cases across the globe, resulted in the declaration of a global pandemic by the World Health Organization on March 11, 2020; on March 15, 2020, the president of South Africa declared a national state of disaster (*2*). That declaration resulted in the establishment of a National Coronavirus Command Centre and national implementation of nonpharmaceutical prevention measures, such as closures of nonessential private industries, school closures, restrictions on public gatherings, social distancing, citizen curfews (including household confinements), and restrictions on international and domestic interprovincial travel.

Accurate and timely data are essential to stem an outbreak. As COVID-19 cases increased during the first weeks of the pandemic in South Africa, the National Institute of Communicable Diseases (NICD) requested assistance from the US Centers for Disease Control and Prevention South Africa (CDC-SA) with data capture from laboratory-confirmed cases because the number of paper forms received exceeded existing capacity. A measure of the success of this support was reflected in the ability of the government of South Africa (GOSA) publishing daily COVID-19 related statistics in the media.

Although initial requests for support were received from the NICD, these requests were closely followed by similar requests from the National Department of Health (NDoH) Coronavirus Command Council. As a result, CDC-SA was approached to extend epidemiologic and surveillance support to the national level, especially to the provincial levels buckling under the strain of providing timely and accurate COVID-19 data to the NDoH Coronavirus Command Council (Appendix).

CDC-SA has a long history of providing public health support to GOSA through the US President’s Emergency Plan for AIDS Relief (PEPFAR). Through providing HIV and tuberculosis technical assistance and support of direct service delivery, CDC-SA has developed a close working relationship with NICD and the national and provincial departments of health. CDC-SA staff members bolstered multiple elements of the COVID-19 response, adding to the public health response and surveillance capacity of GOSA. For example, CDC-SA leveraged its existing relationship with the NICD to strengthen national COVID-19 surveillance by supporting data capture and file organization, data quality reviews, and data analytics, laboratory strengthening, and COVID-19 guidance development and review. In addition, CDC-SA supported GOSA with the deployment of senior CDC staff epidemiologists at the national and provincial government levels.

CDC-SA was nimble in providing support when the country was operating under GOSA-authorized COVID-19 lockdown restrictions. CDC-SA supported the NICD by sending a team of staff members, including senior epidemiologic and clinical experts, to support the NDoH Coronavirus Command Council. On March 5, 2020, CDC-SA also deployed a group of epidemiologists and surveillance experts to 7 provinces across South Africa. On March 23, 2020, CDC-SA deployed its first epidemiologist to the KwaZulu-Natal Province, where the COVID-19 outbreak was first identified in South Africa. Provincial support varied depending on need and included providing assistance with developing data management and reporting systems, providing up-to-date clinical guidance and relevant protocol guidelines related to isolation and quarantine regimens, providing geographic outbreak data, and supporting hotspot mapping for response targeting, cluster outbreak investigations, and myriad other technical areas dependent on the needs of the provincial departments of health. Central to this support was the notion of skills transfer from CDC-SA staff to GOSA staff. GOSA staff continue to incorporate these lessons learned by ensuring daily data are disseminated for public circulation.

Internal and external stakeholder coordination was critical to the successful and timely deployment of multiple CDC-SA staff. Identifying a senior CDC-SA staff member to coordinate these efforts enabled senior-level engagement across GOSA and their US Government counterparts. Identifying the needs within each province highlighted the varying epidemiologic and surveillance capacity gaps across the different provinces. Provincial deployers provided various iterations of the following support for a duration of 3–6 weeks per deployment:

- Ensuring case surveillance and timely and complete reporting of cases and contacts to NICD and NDoH
- Assisting with collecting, entering, and managing COVID-19 case report data; data cleaning; providing epidemiologic guidance in the analysis and interpretation of epidemiologic data; and responding to requests from NICD and NDoH (e.g., providing the latest guidance updates on contact tracing and testing)
- Supporting the compilation of various surveillance reports (e.g., death surveillance)
- Providing provinces with technical assistance to evaluate the readiness of health systems and supply chain for key medical equipment, including personal protective equipment, oxygen, and ventilators
- Providing technical support to review and assist in drafting key COVID-19 outbreak and laboratory testing guidance documents and policies, including refining outbreak case definitions
- Providing outbreak mitigation support and recommendations for infection prevention and control initiatives at multiple types of public facilities, including hospitals and correctional facilities.
- Supporting the development of clinical, health promotion, and training guidance and policies
- Advocating for and encouraging the transition from a contact tracing and containment focus to community mitigation strategies as the outbreak progressed throughout 2020

In total, 47 CDC-SA staff joined national and provincial government response teams. This valuable support provided the much-needed capacity to GOSA and enabled skills transfer and sustainability of these skills to GOSA counterparts. Tools, dashboards, and training and health promotion materials continue to be used in the management of COVID-19 in South Africa through support from CDC

Communication between provincial government officials in South Africa with their US Government counterparts was essential. A total of 21 provincial technical support deployments occurred over the 6-month period during March–September 2020 (Table 1). The communication levels and types (Table 2) were an essential aspect of engaging all key stakeholders in CDC-SA staff deployments, thereby ensuring alignment with the larger GOSA COVID-19 response effort and US diplomatic priorities and ensuring the safety and security of those deployed. In addition, support provided highlights the ability of CDC to be responsive to requests from GOSA.

**Table 1:** CDC-SA COVID-19 response support, South Africa, March–September 2020*

Deployment type	No. personnel
Provincial support	21
NICD data entry, data analytics	9
National Command Center technical assistants	3
Driver support team	6
Logistics support team	5
CDC-SA management support	3

*CDC-SA, US Centers for Disease Control and Prevention–South Africa; NCID, South Africa National Institute of Communicable Diseases.

**Table 2:** CDC-SA COVID-19 communications response, South Africa, March–September 2020*

Agency	Communications
National Department of Health	• Head of Department of the Provincial Department of Health: notification of the location, scope of work, and name of each CDC-SA staff member • NICD: notification of the location, scope of work, and name of each CDC-SA staff member to ensure provincial and national cooperation of COVID-19 data flow • Provincial NICD points of contact: notification of the location, scope of work, and name of each CDC-SA staff member to ensure provincial and national cooperation of COVID-19 data flow
US government communications	• Notification to US senior leadership within South Africa, including the nature and scope of support provided by CDC-SA • Notification to provincial consular offices, with formal communications to Consul Generals under which the 7 provinces fall • Weekly calls with consular staff for general COVID-19 updates
CDC-SA deployment team	• Nightly (Monday–Friday) check-in calls for all deployers with the CDC-SA coordination team over the course of the 6-month deployment. These calls provided not only an update on the ever-changing global COVID-19 clinical guidance but provided an essential psychological support to deployers • Coordination calls with the logistic teams, including the driver support team. Because of the challenging nature of the times, including the severe nature of the national lockdown requirements, those engagements became essential in navigating many unknown aspects of travel, personal protective equipment requirements, COVID-19 exposures, and, most important, collegial support

*CDC-SA, US Centers for Disease Control and Prevention–South Africa; NCID, South Africa National Institute of Communicable Diseases.

Nightly check-in meetings with deployers became a fundamental element of the technical support provided to deployers. The check-ins also served as critical psychosocial support during a high-risk and uncertain time. Included in these calls were key CDC-SA clinicians and surveillance and laboratory technical specialists, who provided up-to-date guidance on the technical aspects of the deployments. In addition, CDC-SA provided routine updates to the South Africa PEPFAR Coordinating Office and PEPFAR South Africa, which were implementing partners working in close coordination on COVID-19 efforts with provincial governments.

These deployments in the setting of COVID-19 raised multiple logistical challenges, particularly the provincial deployments. In early March 2020, all provincial travel in South Africa was prohibited. CDC-SA expeditiously coordinated with a CDC-SA team of drivers to transport epidemiologic and surveillance specialists to and within 7 of the 9 provinces across South Africa. All drivers were certified as essential workers and obtained permission to transport CDC-SA staff. The provision of this essential service by the CDC-SA driver team enabled the prompt deployment of CDC-SA staff members during extremely challenging times, including having no operational airports, dealing with interprovincial law enforcement checkpoints, risking exposure to SARS-CoV-2, and limited availability of hospitality services and accommodations because of the national lockdown.

The ability to respond rapidly and provide support was largely thanks to the efforts of the CDC-SA logistics team. The logistics team was responsible for organizing crucial land transportation to and from the deployment sites for the teams, with transport often occurring outside of curfew, requiring the necessary GOSA approvals.

The success of the rapid scale-up of the CDC-SA deployments can be attributed to the leadership, management, and technical skills of the different team members. In addition, all deployments were based on the notion of volunteerism, which included navigating the unknown risks in the early stages of the pandemic in the first few months of 2020. This aspect was acknowledged by GOSA in various communications to CDC.

By mid-September 2020, the first wave of the COVID-19 pandemic in South Africa had passed. As a result, CDC-SA staff returned to their core tasks of providing technical HIV and tuberculosis support to GOSA. GOSA’s capacity to provide epidemiologic and surveillance support has grown stronger in the 18 months since the onset of the pandemic, with demand for support from CDC-SA decreasing in frequency.

Currently, the CDC-SA continues to provide much-needed support around HIV and tuberculosis while, simultaneously and proactively integrating dimensions of COVID-19 best practices into the daily work of our implementing partners, where possible and as needed. These include the following lessons learned:

1. Rapid, accurate data on status of the outbreak for developing policy and informing leadership, the public health and clinical communities, and the general public. Providing the tools and expertise to help accomplish robust data dissemination is critical to a successful support effort.
2. Up-to-date guidance and recommendations are critical and often challenging, given insufficient data and expertise and changing situations. Providing expertise and support to complement the local expertise in developing guidance and recommendations is important to supporting the response.
3. Substantial physical and emotional strain afflicts staff responding to a pandemic. Providing personnel support to decrease the workload and emotional support to deal with the stress are important to assisting the response.
4. A pandemic response is complex and ever-changing, making frequent (e.g., daily) communication among participants essential to coordinating a successful support effort.

The need for CDC-SA national and provincial-level deployments is continuously reassessed, taking into consideration national and provincial COVID-19 indicators and ongoing consultation with GOSA. However, CDC-SA remains on standby to respond to the pandemic as the need arises, having gathered greater experience through lessons learned on pandemic response.

Appendix Additional information about use of epidemiology surge support to enhance robustness and expand capacity of SARS-CoV-2 pandemic response, South Africa.
