# Supplementary material for: Use of Epidemiology Surge Support to Enhance Robustness and Expand Capacity of SARS-CoV-2 Pandemic Response, South Africa
Source: Emerg Infect Dis. 2022 Dec;28(Suppl 1):S177–80. doi: 10.3201/eid2813.212522 (PMC9745251; doi:10.3201/eid2813.212522)
Supplement: Appendix — Additional information about use of epidemiology surge support to enhance robustness and expand capacity of SARS-CoV-2 pandemic response, South Africa. [file 21-2522-Techapp-s1.pdf]

Article DOI: https://doi.org/10.3201/eid2813.212522

Use of Epidemiology Surge Support to Enhance Robustness and Expand Capacity of SARS-CoV-2 Pandemic Response, South Africa

Appendix

Support Provided by CDC Staff to District, Provincial, and National Governments in South Africa

- Daily data capturing and contact tracing.
- Developed a Provincial Funeral Register and trained parlor and health workers on its use in 5 districts.
- Participated in Provincial COVID-19 War Room and Incident Response Task Team and provide epidemiologic support.
- Participated in investigating, analyzing Covid-19 mortality statistics – including supporting clinical audits, cluster outbreaks [in hospitals, retails, homes.] investigations etc.
- Supported the development of a provincial Covid-19 ‘resurgence monitoring and development of guidelines on management of mortal human remains. Plan
- Participated in a provincial Covid-19 risk communication and community engagement (RCCE) epidemiology, data management, surveillance, clinical governance subgroup.
- Compilation and presentation of the provincial COVID-19 Daily Situation Reports.
- Conducted rapid risk assessments to inform local resurgence plans and support district readiness plans.

- Provided surveillance support focused on outbreaks. For example, in correctional facilities and schools.
- Provided standard reporting templates to SAG staff, which were mostly based on using Microsoft Excel.
